# Vitamin E Analogs as Radiation Response Modifiers

**DOI:** 10.1155/2015/741301

**Published:** 2015-08-20

**Authors:** Pankaj K. Singh, Sunil Krishnan

**Affiliations:** Department of Radiation Oncology, The University of Texas MD Anderson Cancer Center, Houston, TX 77030, USA

## Abstract

The potentially life-threatening effects of total body ionizing radiation exposure have been known for more than a century. Despite considerable advances in our understanding of the effects of radiation over the past six decades, efforts to identify effective radiation countermeasures for use in case of a radiological/nuclear emergency have been largely unsuccessful. Vitamin E is known to have antioxidant properties capable of scavenging free radicals, which have critical roles in radiation injuries. Tocopherols and tocotrienols, vitamin E analogs together known as tocols, have shown promise as radioprotectors. Although the pivotal mechanisms of action of tocols have long been thought to be their antioxidant properties and free radical scavenging activities, other alternative mechanisms have been proposed to drive their activity as radioprotectors. Here we provide a brief overview of the effects of ionizing radiation, the mechanistic mediators of radiation-induced damage, and the need for radiation countermeasures. We further outline the role for, efficacy of, and mechanisms of action of tocols as radioprotectors, and we compare and contrast their efficacy and mode of action with that of another well-studied chemical radioprotector, amifostine.

## 1. Introduction

Scientists around the world have known of the deleterious and potentially life-threatening effects of human exposure to ionizing radiation (IR) for over a century. Whereas ionizing radiation can be put to therapeutic use in clinical diagnostic radiology and radiation oncology, the possibility still exists of intentional or unintentional exposure of people to radioactive material [1]. The increasing use of ionizing radiation for the treatment of cancer and efforts to escalate the dose of radiation to achieve greater tumor control or to retreat areas near previously treated areas raise the possibility of damaging sensitive and critical normal tissues adjacent to the tumor. On the nonclinical side, the risk of exposure has increased in recent years with the proliferation of nuclear weapons and expansion of nuclear power plants, which collectively pose a risk of unexpected encounters with small or even potentially lethal doses of radiation. In fact, at least 105 (civilian and military) nuclear reactor accidents were documented globally between 1952 and 2015 that resulted in the loss of human life or property [2]. Collectively, these concerns provide the impetus to identify, develop, and validate potential strategies to protect normal tissues from the harmful effects of radiation. In this review, radiation countermeasures are defined as use of an agent to minimize the deleterious effects of radiation therapy by administering the compound after the radiation exposure has occurred, regardless of toxicity. The term “radioprotection” refers to preexposure prophylaxis from radiation-induced side effects, which is possible only when the radiation exposure is known or planned, as is the case in clinical settings. A more appropriate term to describe postexposure prophylaxis is radiation mitigation, wherein the agent accelerates the repair or recovery of damaged tissues and organs.

By definition, then, accidental exposure warranting radioprotection typically occurs in uncontrolled environments and could be a consequence of intentional or unintentional contact with radiation. The widespread use of radioactive sources for energy generation, diagnostic and therapeutic medicine, engineering and construction, tracing pollutants, and sterilization of food and other products increases the likelihood of such an encounter. From a national security perspective, the abundance of radioactive sources that are often loosely maintained and inventoried worldwide and the potential for unlawful use of these materials with only marginal barriers to access underscore the need for preparedness for exposure of military personnel and first responders to radiological and nuclear devices [3]. To address these concerns, the Office of Science and Technology Policy and the United States Department of Homeland Security have identified radiation countermeasure development as the highest priority for preparedness against a potential bioterrorism event.

Around seven decades ago Patt et al. reported the first instance of in vivo protection against the ionizing radiation by an organic substance, where a sulfur-containing amino acid, cysteine, was shown to protect rats from the lethal dose of X-rays [4]. Since then, researchers around the world have screened a plethora of chemical and biological compounds for their potential use as novel radiation countermeasure agents. To date, only the synthetic thiol compound, amifostine, has been approved by the United States Food and Drug Administration (FDA) for patients undergoing radiotherapy. However, the adverse side effects of amifostine have greatly restricted its use. Therefore, the quest continues to identify and develop effective, nontoxic, stable, biocompatible radiation countermeasures [5]. Among the newer agents being evaluated for their radioprotective efficacy are the various analogs of vitamin E and their derivatives, which have attracted the attention of researchers within the military and elsewhere. Besides the protection afforded against acute exposure to total body ionizing radiation [6–10], vitamin E analogues have been extensively studied for the protection of organs against partial body irradiation [11–17]. Vitamin E family members have also been shown to enhance the tumor killing effect of ionizing radiation [18–23]. This review outlines the current status of these research endeavors and the gaps in knowledge that remain to be addressed in future investigations before the full-fledged development of these compounds into radiation countermeasures. It also highlights the tumor radiosensitizing effect of selected vitamin E family members.

## 2. Effects of Ionizing Radiation

Ionizing radiation possesses sufficient energy to strip electrons out of atoms or molecules to create atoms or molecules with unpaired electrons called free radicals. At sufficiently high doses, ionizing radiation induces ionization events leading to damage to DNA, proteins, or membrane lipids, either directly or indirectly through the intracellular generation of reactive oxygen species (ROS) including superoxide anion, hydrogen peroxide, hydroxyl radicals, peroxide radicals, and other free radicals. Exposing an individual to total body irradiation (TBI) over a brief period initiates a cascade of localized and generalized tissue injuries that manifests as a constellation of symptoms referred to as the acute radiation syndrome (ARS). ARS can be classified into subtypes based on the relative response and sensitivity of organs to radiation. Based on the clinical manifestation, ARS has been subcategorized into three distinct syndromes—hematopoietic, gastrointestinal (GI), and neuro/cerebrovascular [24]. Development of these syndromes depends on the quality of radiation, the quantity of radiation, and the time and rate of radiation exposure [25, 26]. Each of these syndromes manifests with the following: (i) a prodromal phase, which commences during the initial period (within an hour to around a day) after the exposure; (ii) a latent phase, the duration of which depends on the intensity of exposure; and (iii) the illness phase, which has the characteristic features of the syndrome bearing its name. The prodromal phase typically manifests as generalized constitutional symptoms such as nausea, vomiting, malaise, and myalgia. The time to onset of nausea and vomiting correlates directly with the radiation dose that the individual is exposed to. The prodromal phase is followed by the latent phase. During this period, the exposed individual remains relatively symptom-free. The length of the latent phase varies from hours to even a month depending on the intensity of radiation exposure. The hematopoietic syndrome has a longer latency period than the GI syndrome (lasting a few days to a week) or the neurovascular syndrome (lasting just hours in duration). The illness phase appears with the classical clinical symptoms associated with the major organ system that has been injured (bone marrow, intestine, or neurovascular effects).

The hematopoietic syndrome results from total body acute radiation doses of 1.5 Gy or more. This syndrome results from the deterioration in bone marrow function with pancytopenia, the magnitude of which increases drastically with increasing intensity and time of exposure. After radiation exposure, the lymphocytes counts decline fastest, followed by the leukocytes and lastly the erythrocytes. The consequences of this pancytopenia are increased risk of infections, bleeding problems, and anemia. In some instances, this syndrome can be treated and potentially cured with antibiotics to prevent or treat infections, transfusion of blood products to replace depleted blood counts, and growth factors and cytokines to stimulate the bone marrow to produce more blood cells and in extreme cases with bone marrow transplantation.

The GI syndrome typically results from total body acute radiation doses greater than approximately 10 Gy, but some clinical symptoms can appear after doses as low as 6 Gy [27]. The clinical symptoms of GI syndrome include malaise, nausea, vomiting, anorexia, electrolyte imbalance, dehydration, abdominal pain, and secondary infections resulting from destruction of the GI tract and bone marrow [28]. The chances of surviving this syndrome are extremely low, with life expectancy less than 2 weeks [27].

The neuro/cerebrovascular syndrome typically results from exposure to doses greater than 30 Gy, although some clinical symptoms may be identified at doses as low as 20 Gy. This syndrome has a very short latency period and manifests as dizziness, headache, or decreased level of consciousness that develops within minutes to a few hours. The absence of nausea and vomiting distinguishes the neuro/cerebrovascular syndrome from the GI syndrome. No viable prevention or mitigation strategy has been found for the neuro/cerebrovascular syndrome, which is universally fatal.

### 2.1. Radiation-Induced Reactive Oxygen Species

Atoms, molecules, or ions that have unpaired valence electrons are considered free radicals. The unpaired electron makes the free radical highly reactive towards other molecules. Ionizing radiation, which possesses very high energy, breaks the covalent bonds of molecules to generate free radicals. These free radicals interact with each other and with oxygen to mediate radiation injury [24]. The radiation-induced dissociation, radiolysis, of water generates reactive oxygen species (ROS), including superoxide, hydrogen peroxide, and hydroxyl radicals. Protective enzymes like superoxide dismutase (SOD), catalase, and peroxidase play an important role in keeping the intracellular ROS within the tolerable limits. SOD, which is a metalloprotein, catalyzes the toxic superoxide radicals to less damaging species like molecular oxygen or hydrogen peroxide. For Cu SOD, this dismutation of superoxide involves two half reactions; in the first half reaction, superoxide radicals are converted to molecular oxygen while Cu(II)SOD is reduced to Cu(I)SOD and, in the second half reaction, superoxide radicals are converted to hydrogen peroxide while Cu(I)SOD is oxidized back to Cu(II)SOD. Hydrogen peroxide, in turn, is converted to oxygen and water by the enzyme catalase. Hydrogen peroxide interacts with the asparagine-147 and histidine-74 amino acids of the active site of Fe(III)catalase causing the transfer of a hydrogen ion between the oxygen atoms. Subsequently, the free oxygen atom coordinates with the active site to form O=Fe(IV)catalase, freeing the water molecule. Later, O=Fe(IV)catalase reacts with another hydrogen peroxide molecule restoring Fe(III)catalase and releasing water and an oxygen molecule.

To counter the surge in ROS, the innate redox homeostatic mechanism in the cell efficiently scavenges free radicals via the antioxidant properties of glutathione. A mismatch between the generation of reactive oxygen species and the scavenging of these species contributes to oxidative stress and downstream events including oxidation of membrane lipids, proteins, and DNA. Intracellular signaling events initiated by ROS include mitochondrial release of cytochrome c which, in turn, activates the intrinsic apoptotic cascade and causes cell death.

Quite naturally, the recognition of radiation-induced ROS as the proximate cause of cellular death has fueled the quest for identification of antioxidant molecules that could serve as potential radiation countermeasures [29, 30]. An obvious solution would be to stimulate the innate intracellular mediators of the antioxidant response (glutathione, thioredoxin, superoxide dismutase, and catalase) that may be overwhelmed by the sudden burst of oxygen free radicals induced by radiation. Alternatively, extrinsic supplementation of these same intrinsic defense mechanisms can result in scavenging of free radicals before they damage critical cellular structures and functions. Lastly, analogs of such intrinsic scavengers administered after the radiation exposure can serve as reducing agents, or newer synthetic molecules that have the same scavenging properties can be custom-designed. Prominent among these strategies is the use of thiol compounds, polyphenols, superoxide dismutase mimetics, and vitamin E analogs, all of which have been investigated as potential radiation countermeasures in recent years [29, 30].

## 3. Radiation Countermeasures

As described earlier, radiation countermeasures are those instituted after radiation exposure has occurred, and they act to minimize or eliminate damage to individual cells, organs, and the person. The efficacy of an agent can be quantified in terms of the dose-modifying factor (DMF) or dose-reduction factor (DRF), term that refers to the ratio of the radiation dose in the presence of the drug that results in a given deleterious biological effect to the radiation dose in the absence of the drug that results in the same biological effect. The major determinants of the DRF of a radiation mitigator are (a) the mechanism of action of the agent, (b) the time and route of administration, (c) the extent of accumulation in the tissue or organ being targeted, (d) the dose of drug, (e) the model system used, (f) the endpoint being evaluated, and (g) the radiation quality (i.e., type and dose rate). Among a variety of such agents identified and tested [29, 31–37], the most promising agent that emerged from a screen of more than 4000 compounds evaluated at the Walter Reed Institute for Research was an aminothiol called amifostine (WR-2721). Amifostine is a thiol-based agent that scavenges free radicals and is less toxic than cysteine and its decarboxylated analog cysteamine. Its DRF for radiation-induced hematopoietic syndrome in mice is 2.7. The history of the development of amifostine as a radiation countermeasure has been extensively chronicled in the literature [10, 30, 38–42]. Key attributes for its use as a radiation countermeasure are its narrow therapeutic window, its need for intravenous administration, and its need for administration relatively soon after radiation exposure [43, 44]. Nevertheless, the lessons learned from evaluating amifostine were that any agent being actively pursued as a radiation countermeasure should be (a) effective more than a few hours after radiation exposure, (b) effective against acute and chronic radiation syndrome, (c) capable of protecting multiple, if not all, organ systems, (d) devoid of significant toxic side effects, (e) rapidly absorbed and distributed throughout the body, (f) suitable for easy administration in a mass casualty situation (oral or subcutaneous being the more acceptable routes), (f) chemically stable for storage and handling in adverse environments, (g) readily available or manufactured easily for scale-up, and (h) inexpensive. To date, no agent has fulfilled all these criteria, but numerous agents are proceeding along the path to eventual designation as a prototype radiation countermeasure. Outlined below is the progress made to date in labeling vitamin E and its derivatives as radiation countermeasures.

### 3.1. Vitamin E

Natural compounds or nutraceuticals with human health benefits are understandably attractive for use as radiation countermeasures because these compounds are found in our diets and are generally considered safe for clinical use as preventive or therapeutic agents. Nutraceuticals are generally thought to be more appropriate for medicinal use than “unnatural” synthetic analogs because nutraceuticals are well tolerated and have negligible toxicity, perhaps even when consumed in large quantities.

**Figure 1:**
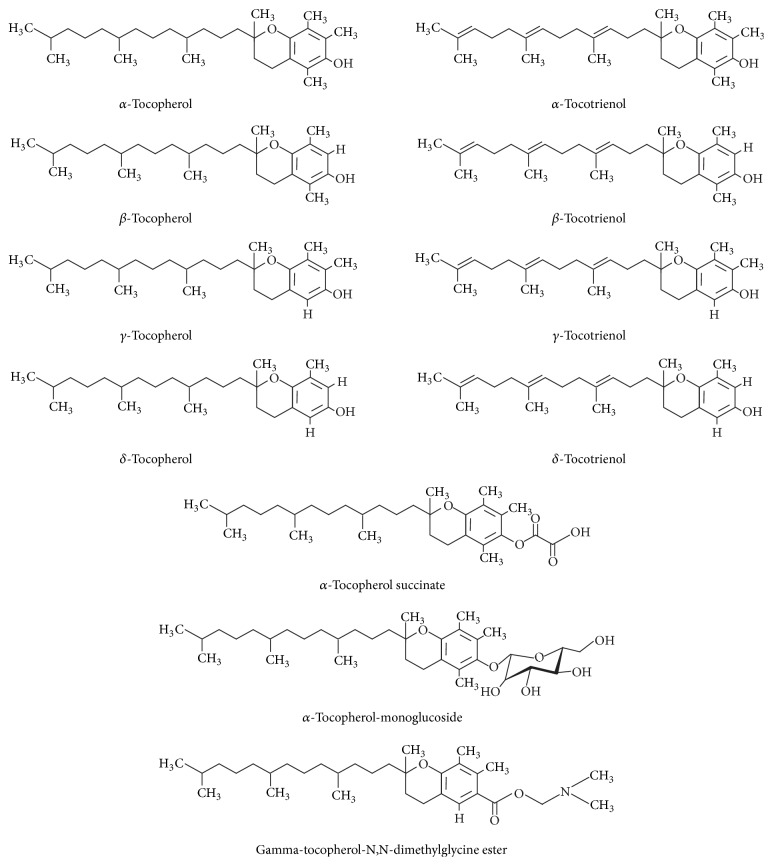
Chemical structures of tocols and their derivatives.

Vitamins are vital nutrients with diverse biochemical functions that are essential for maintaining health. Often sourced from orally consumed dietary natural compounds [45], vitamin E is an essential, fat-soluble nutrient with antioxidant, neuroprotective, and anti-inflammatory properties [46]. Vitamin E is a generic term for all biologically active stereoisomeric compounds of tocopherols and tocotrienols (Figure 1) [47], described further below.

#### 3.1.1. Structural and Physiological Comparison of Tocopherols and Tocotrienols

Both tocopherols and tocotrienols have four analogs (*α*, *β*, *γ*, and *δ*). These eight analogs are collectively known as tocols. Structurally, the tocols consist of a chromanol ring and a 15-carbon tail at the C-2 position. The four analogs of tocopherol and tocotrienol can be distinguished by the number and location of methyl groups on the chromanol rings (Figure 1). The presence of three transdouble bonds in the hydrocarbon tail structurally distinguishes tocotrienols from tocopherols. Tocopherols and tocotrienols are both metabolized via *ω*-hydroxylation initially and then by five cycles of *β*-oxidation [48]. The rates at which the individual vitamin E isoforms are metabolized markedly affect their bioavailability and bioequivalence. By virtue of their differences in structure and conformation, tocopherols and tocotrienols have distinct molecular and therapeutic attributes. Further, analogs of these compounds, which differ in the number of methyl groups, also have different biological activities. Important structural and physiological similarities and differences between tocopherols and tocotrienols are listed in Table 1.

Factors that influence the radioprotective efficacy of tocotrienols and tocopherols include the following: (a) distribution in the lipid bilayer, as tocotrienols are distributed uniformly therein; (b) interaction with the lipid bilayer, in that the chromanol ring of tocotrienols interacts potently with and directly integrates within the lipid bilayer; (c) recycling efficiency, which is higher for tocotrienols [49]; (d) rate of cellular uptake, which is 70 times greater for tocotrienol than for alpha-tocopherol [50]; and (e) rate of absorption after oral administration—tocotrienols, for example, appear in plasma in mice faster than tocopherols because they are absorbed faster by intestinal epithelial cells [51]. Tocotrienols can be metabolized to tocopherols in vivo; however, the rate of conversion varies considerably across species. Although this conversion rate is higher in human than in swine, the amount of tocotrienols converted to tocopherols is quite low, perhaps too low to have any biological implications [52–54].

Supplementation of laboratory animal diets with tocopherol raises serum levels of total cholesterol and low-density lipoprotein, whereas diets fortified with tocotrienol reduce these serum levels [55]. The *α*-tocotrienol compound inhibits 3-hydroxy-3-methylglutaryl-coenzyme A (HMG-CoA) reductase, a regulatory enzyme in cholesterol biosynthesis, whereas *α*-tocopherol either inhibits or stimulates liver HMG-CoA reductase [55–57].

Tocotrienols have been shown to prevent cancer, cardiovascular, and neurodegenerative diseases [58]. Although *α*-tocotrienol has been shown to be neuroprotective in nature [59], *δ*-tocotrienol (DT3) has been found to be effective in targeting prostate cancer stem cell-like populations [60] and pancreatic cancer [61]. *α*-Tocotrienol, but not the tocopherols, inhibits the early activation of c-Src kinase [59] and thereby protects neural cells from glutamate-induced 12-lipoxygenase activation and consequent cell death [62]. Tocotrienols inhibit the expression of vascular endothelial growth factor (VEGF) receptor expression in human umbilical vein endothelial cells, thereby blocking VEGF signaling. Tocotrienols, but not the tocopherols, inhibit tumor-induced angiogenesis when administered orally [63].

Significant amounts of tocopherols and tocotrienols can be isolated from a variety of food sources. The abundance of these tocols differs considerably across different food sources [45, 64]. As noted in Table 2, peanut, wheat, and soybeans have more tocopherols than tocotrienols, whereas oats, barley, palm oil, and rice bran oil have more tocotrienols.

### 3.2. Vitamin E Derivatives as Radiation Protectors

Multiple groups have demonstrated normal tissue radioprotective, tumor radiosensitizing, and single-agent antitumor activity of tocopherols and tocotrienols [18–20, 27, 30, 65–68]. Most of these studies were done with *α*-tocopherol, which is the most abundant analog of vitamin E in human and animal tissues [27, 30, 65]. However, tocotrienol research has gained prominence during the past decade. Subcutaneous administration of all forms of vitamin E analogs at 24 h before radiation exposure has shown protective effects against ARS [69–72]. The most commonly described mechanism of action of these radioprotective agents is their function as antioxidants, which could involve free radical scavenging or catalytic decomposition by enzymes. Antioxidant activity has not been consistent between publications owing to variation in the terms used, including the rate of scavenging, for example, near-diffusion or controlled, and the concentration for effectiveness (free radicals scavenged per mole of an antioxidant). Tocotrienols have shown superior antioxidant activity to tocopherols [73–75], with one report documenting tocotrienols having 1600 times greater antioxidant activity than *α*-tocopherol [49]. However, others have suggested no differences in the antioxidant potential of tocopherols and tocotrienols [76]. The reducing and scavenging activity of tocols undoubtedly depends on the experimental design and the circumstances under which assays are performed. The superior antioxidant efficacy of tocotrienol may result from its unsaturated aliphatic tail, which aids penetration into tissues. Among the tocopherol isoforms, *α*-tocopherol has been shown to have better antioxidant activity than *γ*-tocotrienol, *δ*-tocotrienol, or tocopherol succinate. However, the radioprotective efficacy of these three tocols does not follow the same trend—the radioprotective efficacy of a tocol is not determined solely by its antioxidant activity. The vitamin E derivatives that have shown significant activity after acute exposure to ionizing radiation and have the potential to be further developed as radiation countermeasures are outlined in the following sections.

#### 3.2.1. Tocopherols

Tocopherol was first identified in the 1930s as a dietary ingredient essential for the maintenance of fertility in rats and consequently derives its name from the Greek words “tokos” and “pherein” that together signify the “bearing of offspring” [77]. Tocopherol and its derivatives have been shown to have anticancer effects [68, 78, 79], radiosensitizing potential [18–21], and radioprotective properties in different experimental model systems. Taken together, these reports suggest that tocopherols have the potential to differentially protect normal cells from radiation-induced damage while enhancing the effects of radiation on cancerous cells. The mechanistic underpinnings of this apparent dichotomy and discrepancy are not fully understood and warrant continued investigation. Another feature of the tocopherols that impacts their translational potential is the water solubility and consequently their route of administration and potential for toxicity. Surprisingly, despite sourcing of tocopherols primarily from plants and nature, solubility and toxicity remain potential concerns that warrant continued attention while clinical translation is advanced. Alpha-tocopherol and alpha-tocopherol succinate are water insoluble, whereas alpha-tocopherol-monoglucoside and gamma-tocopherol-N,N-dimethylglycine ester are soluble in water. In the ensuing paragraphs, we will provide an overview of the key attributes of these tocopherols that make them potentially viable countermeasures against accidental ionizing radiation exposure and the emerging evidence of possible radiosensitizing effects of tocopherols in cancer cells.

*(1) Alpha-Tocopherol*. The radioprotective properties of tocols have been demonstrated in several recent reports [72, 80–84]. In one such study, subcutaneous administration of *α*-tocopherol (100 IU/kg body weight) either 1 h before or 15 min after *γ*-irradiation significantly increased the 30-day survival of mice, with DRFs of 1.06 (1 h before) and 1.11 (15 min after) [84]. Higher subcutaneous doses of *α*-tocopherol (400 IU/kg body weight) enhanced the survival of irradiated mice when given 24 h before *γ*-irradiation with ^60^Co at a dose rate of 0.6 Gy/min [72]. Oral administration of *α*-tocopherol significantly reduced the frequency of micronuclei formation and chromosomal aberrations in the bone marrow cells of mice exposed to 1 Gy of radiation [83]. Total body gamma-irradiation of mice followed immediately by administration of *α*-tocopherol results in a surge in the number of hematopoietic colony-forming units in the spleen suggesting that *α*-tocopherol also stimulates recovery or repair processes [85]. Another series of studies suggested that the radiomitigative effects of *α*-tocopherol in mice may result from its ability to enhance cell-mediated immunity [82, 86]. The window of radioprotection for *α*-tocopherol is about 24 h with circulating blood levels of *α*-tocopherol peaking at 24 h and at 4 h. This latter finding suggests that *α*-tocopherol induces the expression of other factors required for radioprotection and that these factors may have a greater radioprotective effect than *α*-tocopherol itself. Several tocopherol analogs subsequently evaluated for their ability to induce cytokines and growth factors that mediate radioprotective efficacy are described below.

*(2) Alpha-Tocopherol-Monoglucoside*. Alpha-tocopherol-monoglucoside or *α*TMG is a water-soluble derivative of *α*-tocopherol (2-(*α*-D-glucopyranosyl) methyl-2,5,7,8-tetramethylchroman-6-ol) where the linear carbon chain of *α*-tocopherol is substituted with a glucopyranosyl moiety [87]. *α*TMG has better antioxidant activity in terms of inhibition of lipid peroxidation [88]; however, its long hydrophobic phytyl side chain constrains its mobility and limits its activity as a free radical scavenger to the cell membrane. Because *α*TMG is soluble in water, it is a better candidate than other forms of vitamin E for development as a radiation countermeasure agent.

In one series of experiments, oral administration of *α*TMG at doses of up to 7 g/Kg body weight was nontoxic in a mouse model, and intraperitoneal administration of *α*TMG (0.6 g/Kg body wt) protected mice from TBI-induced weight loss and death and shifted LD_50(30)_ from 6 Gy to 6.72 Gy [89]. When given before a dose of 2 Gy of radiation, *α*TMG reduced the radiation-induced mortality among embryos of pregnant mice by 75%. Further, *α*TMG protected against both radiation-induced chromosomal damage and radiation-induced formation of thymine glycol. Other investigators have reported that *α*TMG, given 15 min before or a maximum of 30 min after ^60^Co *γ*-irradiation of Swiss albino mice, can reduce the extent of radiation-induced micronucleated erythrocytes and cells with aberrant metaphase [90]. Intraperitoneal injection of *α*TMG (600 mg/Kg body weight) in mice 10 min after ^60^Co *γ*-irradiation was radioprotective, with a DRF of 1.09 [91]. Another group found *α*TMG to protect the hematopoietic recovery of irradiated mice [92] and to mitigate radiation-induced bone marrow damage [93]. In in vitro studies, *α*TMG mediated inhibition of radiation-induced single-strand breaks in plasmids, suggesting that *α*TMG can protect DNA [94]. However, TMG did not protect the DNA of cancer cells in culture [95].

*(3) Gamma-Tocopherol-N,N-Dimethylglycine Ester*. Gamma-tocopherol-N,N-dimethylglycine ester (GTDMG) is a water-soluble derivative of tocopherol and a prodrug of *γ*-tocopherol [96]. GTDMG's major metabolite is 2,7,8-trimethyl-2S-(*β*-carboxyethyl)-6-hydroxylchroman [96]. A recent evaluation of the protective effects of GTDMG in mice showed that it significantly enhanced 30-day survival when given 30 min before or just after irradiation. Giving GTDMG at 100 mg/Kg body weight intraperitoneally to mice 30 min before TBI with 7.5 Gy significantly protected the bone marrow and increased the survival rate of the mice by 70% [97]. Giving the same concentration immediately after irradiation led to survival rates of about 98%, with a DRF of 1.25, and even giving GTDMG at 24 h after irradiation showed significant mitigation effects [97].

*(4) Alpha-Tocopherol Succinate*. Alpha-tocopherol succinate, the hemisuccinate ester derivative of *α*-tocopherol, is more effective than *α*-tocopherol, *α*-tocopheryl nicotinate, and *α*-tocopheryl acetate in terms of promoting differentiation, enhancing apoptosis, and inhibiting proliferation in cancer cells [77] and thus is considered a promising antitumor agent [20, 98–102]. This derivative was also seen to have the opposite effects on normal cells both in vitro and in vivo, protecting them from chromosomal damage and radiation-induced apoptosis and cytotoxicity [20, 103].

In mice, *α*-tocopherol succinate was protective against ARS in a dose-dependent manner; when administered 24 h before irradiation, it can protect mice from acute doses of *γ*-radiation with a DRF of 1.28. In fact, a single dose of TS (400 mg/Kg body weight), given 24 hours before ^60^Co *γ*-radiation exposure, enhanced the survival rate of mice with gastrointestinal ARS by alleviating radiation-induced intestinal injuries and improving overall intestinal health by restoring crypt cellularity. Moreover, irradiated mice treated with *α*-tocopherol succinate had less DNA damage and apoptosis and higher cellular proliferation in the jejunum. This compound also prevented secondary infections by inhibiting bacterial translocation from the gut to the bloodstream in irradiated mice, perhaps by stabilizing the junctional complexes on the cytoplasmic membrane of gut epithelial cells [41], and reduced inflammation in the intestinal tissue [7]. Collectively, these results suggest that *α*-tocopherol succinate protects against gastrointestinal ARS by fortifying and regenerating the denuded intestinal mucosal lining. The *α*-tocopherol succinate has also been shown to modulate the expression of antioxidant enzymes and to inhibit the expression of oncogenes in irradiated mice [104].

In terms of its effects on the hematopoietic system, *α*-tocopherol succinate has been found to reduce neutropenia, thrombocytopenia, and monocytopenia, but not to affect lymphocyte counts, in irradiated mice; it further enhances the number of colony-forming units in the spleen and the cellularity of bone marrow [70] and produces high levels of peripheral blood granulocyte-colony stimulating factor (G-CSF) and keratinocyte-derived chemokine. Antibodies to G-CSF were found to completely neutralize G-CSF in the circulating blood and to abrogate the protective effect of *α*-tocopherol succinate against ARS [8, 40].

Several lines of evidence confirm that G-CSF induces mobilization of bone marrow progenitor cells into systemic circulation [36]. First, treatment with *α*-tocopherol succinate and the hematopoietic cell-mobilizing compound AMD3100, separately or in combination, led to increased numbers of circulating hematopoietic stem cells as measured by flow cytometry [36]. Second, whole blood obtained from mice treated with *α*-tocopherol succinate rescued irradiated mice from ARS and death, whereas irradiated mice that did not receive the transfusion died [34, 105]. Similar improvements in survival were achieved from transfusion of stem cell-enriched peripheral blood mononuclear cells from the *α*-tocopherol succinate-treated mice [105], presumably because the transfused cells serve as a kind of “bridge” therapy until recovery of the innate immune system of the irradiated mice. Notably, transfusion of whole blood or peripheral blood mononuclear cells allows irradiated mice to survive doses of radiation that typically elicit the GI syndrome; histological and immunohistochemical evaluation of jejunal sections from recipient mice revealed inhibition of apoptosis and increased proliferation of the GI mucosa [34]. These effects were also associated with reduced bacterial colonization of other organs, suggesting that transfusion of cells mobilized with *α*-tocopherol succinate (compared with control-mobilized cells) preserved the intestinal barrier in irradiated mice [34]. The magnitude of induction of G-CSF, and the radioprotective effect, seems to depend on the dose of *α*-tocopherol succinate. The temporal profile of G-CSF production induced by *α*-tocopherol succinate, which peaks at 24 h after administration, also corresponds to the optimal radioprotection noted when *α*-tocopherol succinate is given 24 h before TBI. Theoretically, then, G-CSF could be a biomarker of the radioprotective effects of *α*-tocopherol succinate, supplementing the postexposure monitoring of leukocyte counts to gauge efficacy.

Indeed, the correlation between G-CSF production and radioprotective efficacy has been well described [106–109]. Extrinsic administration of G-CSF increases survival in lethally irradiated mice via faster induction of neutrophil recovery. G-CSF protects mice from the detrimental effects of low doses, but not high doses, of ionizing radiation. Potential advantages of using *α*-tocopherol succinate rather than G-CSF for cytokine therapy are its low cost and ease of storage and administration in a mass casualty situation.

*(5) Radiosensitizing Effects of Tocopherols*. The tumor radiosensitizing effect of tocopherol was first reported in murine neuroblastoma cells where alpha-tocopherol acetate was shown to enhance the effect of X-ray treatment [18]. Later alpha-tocopherol succinate was found to enhance the effect of gamma-radiation in the same neuroblastoma cells [19]. Further alpha-tocopherol succinate was found to differentially increase the radiation-induced chromosomal damage in human cervical cancer cells while protecting normal human fibroblast cells from the deleterious effects of radiation [20]. Alpha-tocopherol succinate increased the length of delay in radiation-induced mitotic accumulation in human cervical cancer (HeLa) cells and ovarian cancer (OVGI) cells but not normal fibroblast (GM2149, AG1522, and HF19) cells [21]. Similarly, administration of *α*TMG immediately after exposure of tumor-bearing mice to gamma-radiation protected the normal cells but not the cancer cells (fibrosarcoma) from development of radiation-induced DNA strand breaks [22]. Alpha-tocopherol succinate treatment enhanced the induction of apoptosis by radiation in MCF-7 breast cancer cells [23]. Even though the mechanism of radiosensitization by tocopherols has not been studied in detail, there are some suggestions that activation of Fas signaling pathway [110], inhibition of angiogenesis [78], inhibition of protein kinase C activation leading to caspase 3 activation [98], inhibition of the DNA binding activity of NF*κ*B [111], and downregulation of c-myc and H-ras [112] could play a role in the observed radiosensitization in a variety of cancer cells. Collectively, these reports of tumor radiosensitization, when coupled with the body of evidence suggesting normal tissue radioprotection, offer the tantalizing prospect that tocopherols can simultaneously protect normal tissues from the deleterious effect of radiation and sensitize cancer cells to radiation therapy.

#### 3.2.2. Tocotrienols

Dunphy and coworkers first reported the discovery and extraction of tocotrienol from rubber in 1964 [113]. It was many years later that tocotrienols were noted to have cholesterol lower properties that garnered the interest of the biomedical community [73]. Tocotrienols were particularly exciting as antilipidemic agents because they were derived from natural and plant sources. Accordingly, the estimated no-observed-adverse-effect-level (NOAEL) for rats was found to be relatively high at 120–130 mg/Kg body weight/day [114]. More recently, there has been a shift in focus from lipid lowering to anticancer properties of tocotrienols. We highlight the normal tissue radioprotective properties of tocotrienols and underscore the potential for radiosensitization of tumors by tocotrienol in the following paragraphs.

*(1) Gamma-Tocotrienol*. Gamma-tocotrienol or *γ*T3 has potent antioxidant properties as well as inhibiting HMG-CoA reductase, similar to statins [115]. When administered at doses of 100 mg/Kg body weight and 200 mg/Kg body weight at 24 h prior to ^60^Co radiation, *γ*T3 protected mice from death after radiation doses as high as 11.5 Gy, with a DRF of 1.29. This effect was associated with increased numbers of reticulocytes, neutrophils, monocytes, and platelets in the peripheral blood [69], suggesting faster hematopoietic recovery. There was an associated increase in hematopoietic progenitors, colony-forming cells, and regenerative microfoci of myeloid and megakaryocytic cells [116]. Also noted were higher cellularity in the bone marrow and a decreased frequency of micronucleated erythrocytes. Consistent with the notion that hematopoietic cell preservation and recovery could be explained by changes in cytokines and growth factors, serum G-CSF levels increased within 12–24 h after *γ*T3 administration before returning to baseline levels by 48 h [117]. Paralleling this increase in G-CSF, albeit at a lower level and peaking earlier than G-CSF, was an increase in interleukin (IL-6). These findings left to the conclusion that *γ*T3 effectively mobilizes hematopoietic progenitors in the peripheral blood, thereby enhancing its radioprotective action [118]. It has been shown that neutralization of G-CSF by the administration of antibody abrogates the radioprotective efficacy of *γ*T3 [119]. Independent of G-CSF, in a proposed alternative mechanism of action, *γ*T3 inhibits the hydroxy-methyl-glutaryl-coenzyme A reductase (HMGCR) enzyme and thereby modulates the expression of thrombomodulin to enhance hematopoietic recovery after total body radiation exposure [120]. Further, induction of G-CSF by *γ*T3 was able to mobilize the progenitor cells [121], which mitigated the deleterious effect of gamma-radiation [121].

Aside from its effects on cytokines and hematopoietic cells, *γ*T3 also reduces radiation-induced oxidative stress within blood vessels, which was readily reversed by mevalonate (the by-product of HMG-CoA metabolism by HMG-CoA reductase) [122]. Because HMG-CoA reductase inhibitors also upregulate endothelial nitric oxide synthase, one team treated mice with *γ*T3 and evaluated vascular endothelial peroxynitrite production by oxidation of nitric oxide. They found that *γ*T3 reduced vascular peroxynitrite production (the oxidation product of nitric oxide) and protected endothelial cells from apoptotic death after radiation exposure [123]. Further mechanistic studies revealed that *γ*T3 decreased transcription of the guanosine triphosphate cyclohydrolase 1 (GTPCH) feedback regulatory protein gene* GFRP* in endothelial cells, thereby releasing the feedback inhibition of GTPCH and increasing the biosynthesis of tetrahydrobiopterin. *γ*T3 also reversed the decrease in tetrahydrobiopterin in the lungs induced by irradiation [123], protected the mice from GI injury, and accelerated the recovery of soluble markers of endothelial function [122]. Further, *γ*T3 has been shown to protect the intestinal cells from the acute dose of radiation by increasing the expression of antiapoptotic factors and decreasing the expression of proapoptotic factors at the transcriptional as well as translational level [124].

Drawing on several preclinical and clinical reports of the efficacy of combining the phosphodiesterase inhibitor pentoxifylline with vitamin E as a strategy to reduce or reverse late radiation-induced cardiac, pulmonary, intestinal, osseous, and dermal fibrosis [80, 125, 126], investigators have explored the combination of *γ*T3 with pentoxifylline to mice from ARS. Indeed, the combination of *γ*T3 and pentoxifylline significantly improved the survival of irradiated mice after exposure to doses as high as 12 Gy by increasing the number of bone marrow colony-forming units and spleen colonies and hastening platelet recovery [127]. However, the combination treatment was no more effective than *γ*T3 alone in terms of protecting against GI injury or reducing vascular peroxynitrite production [127]. Advancement of *γ*T3 as a radiation countermeasure for human use will require documentation of its pharmacokinetics, pharmacodynamics, and radioprotective efficacy in nonhuman primate models.

*(2) Delta-Tocotrienol*. Like *γ*T3, delta-tocotrienol (*δ*T3) also has potent antioxidant properties that can be exploited for radioprotection. When administered as a single subcutaneous dose 24 h before ^60^Co *γ*-irradiation, *δ*T3 at 150 mg/Kg body weight and at 300 mg/Kg body weight protected mice with respective DRFs of 1.19 and 1.27 [128]. Moreover, the higher dose of *δ*T3 reduced radiation-induced cytopenia and hastened hematopoietic recovery. Radioprotective efficacy has been documented from doses ranging from 18.75 mg/Kg body weight to 400 mg/Kg body weight [128, 129]. When administered 2 h after radiation, the DRF for 150 mg/Kg body weight of *δ*T3 was observed to be 1.1 [128]. Favorable pharmacokinetic features of *δ*T3 include a plasma *C*_max_ of 195 *µ*M (*C*_max_) at *T*_max_ of 1 h after injection and plasma clearance 12 h after injection [130]. Mechanistic studies suggest that *δ*T3 strongly inhibits the activation of caspases 8, 3, and 7 and stimulates autophagy-related expression of beclin-1 in irradiated bone marrow cells [130]. In irradiated mice, *δ*T3 was found to increase hematopoietic cell survival, regenerate hematopoietic microfoci and lineage^−^/Sca-1^+^/c-Kit^+^ stem and progenitor cells in the bone marrow, and protect CD34^+^ cells [129]. G-CSF plays a pivotal role in mobilizing progenitor cells. Like other vitamin E analogues, *δ*T3 also induces a high titer of serum G-CSF in mice and facilitates the mobilization of progenitor cells from the bone marrow to the peripheral blood. G-CSF induction by *δ*T3 has been shown to protect mice from ionizing radiation; and conversely the administration of an antibody that neutralizes G-CSF in *δ*T3-treated animals abrogates the radioprotective efficacy of *δ*T3 [131]. Besides the role of G-CSF in *δ*T3-mediated radioprotection, it has been shown that *δ*T3 bestows protection to mice and hematopoietic CD34+ cells from radiation injury by suppressing the radiation-induced microRNA-30 (mir30) and IL-1*β*-induced NF*κ*B/miR-30 signaling pathway [132].

Mechanistically, *δ*T3 is thought to induce extracellular signal-related kinase 1/2 phosphorylation, to upregulate mammalian target of the rapamycin and induce phosphorylation of its downstream effector 4EBP-1, to activate the mRNA translational regulator eIF4E and ribosomal protein S6, and to enhance DNA double-strand break repair, as revealed by decreased numbers of *γ*-H2AX foci. The antioxidant properties of *δ*T3 also contribute to its ability to protect neuronal cells from glutamate toxicity [130, 133].

*(3) Radiosensitizing Effect of Tocotrienol*. In comparison to tocopherols, there are fewer reports demonstrating the radiosensitizing effect of tocotrienols. The initial motivation for exploring radiosensitization by tocotrienols was the demonstration that they have potent proapoptotic activity [134–137]. Kumar and coworkers noted for the first time that *γ*T3 radiosensitizes prostate cancer in a murine model [66]. Subcutaneous injection of *γ*T3 (400 mg/Kg body weight) 24 h before gamma-irradiation was found to reduce the size of tumors by 40%. Higher lipid peroxidation in the tumor was observed acutely (on the 4th day, 150%) as well as later on (on the 24th day, 62%) in the *γ*T3 and radiation treatment group as compared with the control group [66].

### 3.3. Amifostine versus Tocols as Radioprotectors

**Figure 2:**
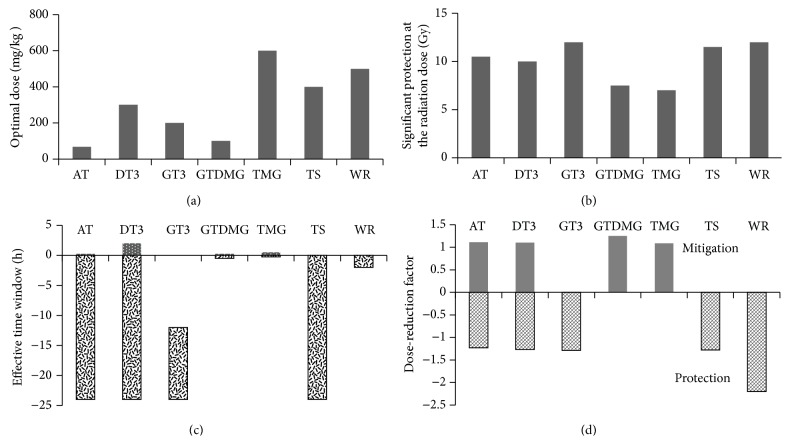
Comparison of tocols and their derivatives with amifostine: (a) comparison of optimum dose, (b) significant protection at highest radiation dose, (c) effective time window for protection and mitigation, and (d) dose-reduction factor for protection and mitigation. Alpha-tocopherol (AT), delta-tocotrienol (DT3), gamma-tocotrienol (GT3), gamma-tocopherol-N,N-dimethylglycine ester (GTDMG), alpha-tocopherol-monoglucoside (TMG), alpha-tocopherol succinate (TS), and amifostine (WR).

The phosphorothioate amifostine was the first drug approved by the U.S. Food and Drug Administration for the prevention of radiation-induced salivary-gland damage and xerostomia in patients with head and neck cancer [10]. Amifostine scavenges free radicals in the intracellular milieu when hydrolyzed by alkaline phosphatase to its active metabolite WR-1065; this effect is stronger in normal tissues because of their relative abundance of alkaline phosphatase and higher pH compared with tumor tissues [138]. Amifostine induces hypoxia via increased use of oxygen and condensation of DNA [139]. DRFs of 2.7 for the hematopoietic syndrome and 1.8 for the GI syndrome in mice have been reported for amifostine administered intraperitoneally at 500 mg/Kg body weight [30]. Even though amifostine has not had particularly promising effects in nonhuman primates, it is still considered the standard against which to compare other radioprotective agents. As such, amifostine has a time window for radioprotection that is quite short (15 min before irradiation); tocols, in contrast, have a wider time window for administration. The radioprotective efficacy of tocols and their derivatives is reasonable, with DRFs ranging from 1.2 to 1.3 (Figure 2). Tocols have been shown to ameliorate radiation-induced hematopoietic as well as GI syndromes. In contrast, enthusiasm for the clinical use of amifostine is dampened by the high incidence of hypotension, especially when administered intravenously at the maximum tolerated dose [140]. Tocols, on the other hand, are well tolerated and have a comparatively higher therapeutic index. A unique attribute of tocols as radioprotectors is their ability to induce high levels of circulating G-CSF, which in turn stimulate hematopoietic recovery. Finally, the possibility that tocols might protect normal tissues from radiation while sensitizing tumors to radiation makes them particularly attractive for clinical use.

### 3.4. Clinical Translational Challenges

As with any formulation being designed for eventual clinical application, the use of tocols as radiation response modifiers requires standardization and optimization of the synthesis, purification, characterization, and analysis of the tocol. Whereas tocotrienols are potentially more efficacious as radiation response modifying agents, their bioavailability is limited and varies considerably based on the route of administration [141]. Their significantly shorter circulatory half-life than tocopherol necessitates larger and more frequent dosing of tocotrienols compared to tocopherols. One explanation for the poor bioavailability is that tocotrienols have a lower affinity for *α*-tocopherol transfer protein (ATTP) and are consequently metabolized by the liver and excreted in the bile, significantly reducing their circulatory half-life [142]. Efforts to improve bioavailability have centered on the use of nanoemulsions, nanoparticulate formulations, or custom synthesis of analogs that bind more efficiently to ATTP. Emulsification, typically with Tween 80, increases absorption of tocotrienols administered subcutaneously [119] and leads to higher plasma concentrations. Oral formulations of tocotrienol with greater bioavailability can be synthesized using hydrophilic polymers like cyclodextrin [143] and intravenous delivery and biodistribution can be enhanced by entrapping tocotrienol in multilamellar vesicles [144]. Lastly, recognizing that docking of alpha-tocopherol to its binding pocket on ATTP requires a flexible tail, a recent study demonstrated that substituting the tri-dienyl chain of the rotationally restricted and rigid farnesyl tail of tocotrienol with mono- or di-dienyl chains gives it sufficient conformational dexterity to bind avidly to the ATTP docking site [145]. In turn, this results in enhanced transport of tocotrienol from the liver to the bloodstream and greater circulatory half-life without adversely affecting antioxidant properties. Clearly, tocols with good bioavailability and excellent safety/toxicity profiles are a prerequisite for clinical translation. Equally importantly, the plasma and tissue concentrations of tocotrienol need to be evaluated [146, 147] to verify that circulating tocol levels correlate with tissue penetration to achieve the desired effects in vivo.

## 4. Conclusions

Radioprotectants are particularly beneficial for individuals exposed to greater risks of accidental radiation exposure, such as first responders and military personnel. Radiation mitigators have some clinical utility when civilians accidentally exposed to radiation are promptly evacuated from sites of radiological fallout. Vitamin E and its derivatives have the potential to serve as both radioprotectors and radiomitigators based on their ability to induce G-CSF, to mobilize hematopoietic precursors from the bone marrow into peripheral circulation, and to accelerate hematopoietic recovery in animal models. Although attention has been focused recently on the lack of efficacy of tocopherol as a chemopreventive agent for prostate cancer [148] or for preventing cardiovascular events [149], it is reasonable to assume that this lack of efficacy does not automatically extend to all tocols and that in fact specific forms of vitamin E such as *α*-tocopherol succinate and tocotrienols may have potent clinical benefits via mechanisms distinct from those of *α*-tocopherol.

In the case of *α*-tocopherol succinate, its radioprotective efficacy extends beyond mobilization of hematopoietic precursor cells and reversal of cytopenias to protection from GI ARS via inhibition of apoptosis, enhancement of proliferation, fortification of structural integrity, inhibition of bacterial translocation, and regeneration of an intact intestinal villous epithelium. Notably, although exogenous administration of G-CSF achieves many of the same protective effects on hematopoiesis and GI tissues, primarily by mobilizing precursor cells, this comes at considerable cost in terms of bone pain, expense, and difficulty with long-term storage. In contrast, *α*-tocopherol succinate mobilizes hematopoietic cells to protect against exposure to a broad range of radiation doses but does not seem to mitigate radiation-induced effects when given after radiation exposure [41].

The tocotrienols *γ*T3 and *δ*T3 have also shown potent radioprotection activity in preclinical models, and their mechanism of action extends beyond simply increasing G-CSF mobilization of hematopoietic precursor cells and hastened recovery from cytopenia. In the case of *γ*T3, its activity as an inhibitor of HMG-CoA reductase results in upregulation of endothelial nitric oxide synthase, reduction of vascular endothelial peroxynitrite production, and protection of endothelial cells from apoptotic death after radiation exposure. As is true for *α*-tocopherol succinate, *γ*T3 also protects mice from GI injury after irradiation. Its hematopoietic effects are amplified when *γ*T3 is combined with pentoxifylline. In the case of *δ*T3, increased hematopoietic precursor cell survival has been attributed to inhibition of apoptosis and induction of Erk 1/2 phosphorylation, upregulation of mTOR, and enhancement of DNA repair.

Despite these early indications of efficacy, further exploratory research is required to (a) develop biocompatible vehicles and formulations for improved bioavailability, (b) evaluate the safety and tolerability of different formulations, routes of administration, and dosing strategies, (c) decipher the mechanism of action at a molecular level, (d) extend efficacy studies to nonhuman primates, (e) investigate the synergistic effect of tocols with other radioprotectors, and (f) identify noninvasive biological markers of efficacy in humans to confirm delivery of an adequate dose.

Taken together, these promising findings of preclinical radioprotective activity of newer tocols beyond *α*-tocopherol warrant continued evaluation of their pharmacokinetics, bioavailability, pharmacodynamics, tolerability, and efficacy in large animal models. A greater understanding of mechanisms of action may facilitate adoption of newer strategies such as alpha-tocopherol succinate-mobilized cellular therapy as a bridge to recovery of innate hematopoietic cells after radiation injury or design of synthetic analogs based on analysis of structure-function relationships critical for specific activity. Collectively, recent advances in our knowledge of vitamin E derivatives provide a framework for the advancement of such agents as viable radiation countermeasures.

## Figures and Tables

**Table 1:** Salient structural and physiobiological properties of tocopherols and tocotrienols.

Attributes	Tocopherol	Tocotrienol
Abundance	Corn, wheat, and soybeans	Barley, oats, palm, and rice bran

Number of analogs	Four analogs (*α*, *β*, *γ*, and *δ*)	Four analogs (*α*, *β*, *γ*, and *δ*)

Presence of chromanol ring and a 15-carbon tail at the C-2 position	Yes	Yes

Type of hydrocarbon tail	Saturated hydrocarbon tail	Unsaturated hydrocarbon tail (three transdouble bonds present in the hydrocarbon tail)

Interaction of the chromanol ring with the lipid bilayer	Not very efficient	Very efficient

Metabolism	Through *ω*-hydroxylation followed by five cycles of *β*-oxidation	Through *ω*-hydroxylation followed by five cycles of *β*-oxidation

Distribution in lipid bilayer	Not in uniform	Uniformly distributed

Recycling efficiency	Lower	Higher

Cellular uptake rate	Lower	Higher

Antioxidant activity	Very high	High

Effect on serum level of cholesterol	Induce the serum levels of both total and low-density lipoprotein cholesterol	Reduce the serum levels of lipoprotein cholesterol

Effect on 3-hydroxy-3-methylglutaryl-coenzyme A (HMG-CoA) reductase	Either inhibits or stimulates	Always inhibits

Effect on c-Src kinase	Does not inhibit the early activation of c-Src kinase	Inhibits the early activation of c-Src kinase

Effect on tumor-induced angiogenesis	Does not inhibit tumor-induced angiogenesis	Inhibits tumor-induced angiogenesis

Effect on mobilization of progenitor cells	Mobilizes progenitor cells from bone marrow to peripheral blood	Mobilizes progenitors cells from bone marrow to peripheral blood

**Table 2:** Natural sources of different analogs of vitamin E (Source: http://www.tocotrienol.org/sources-of-toco.html).

Sources	Tocopherol (milligram/1000 grams)					Tocotrienol (milligram/1000 grams)				
	Alpha	Beta	Gamma	Delta	Total	Alpha	Beta	Gamma	Delta	Total
Palm oil	152				152	205		439	94	738
Rice barn	324	18	53		395	116		349		465
Barley	350	50	50		450	670	120	120		910
Oat	180	20	50	50	300	180		30		210
Coconut oil	5			6	11	5	1	19		25
Wheat germ	1179	398	493	118	2188	24	165			189
Palm kernel oil	13				13	21				21
Soya bean oil	101		593	264	958					0
Sunflower oil	387		387		774					0
Peanut oil	130		216	21	367					0
Cocoa butter	11		170	17	198	2				2
Olive oil	51				51					0
